# Perspectives on precision cut lung slices—powerful tools for investigation of mechanisms and therapeutic targets in lung diseases

**DOI:** 10.3389/fphar.2023.1162889

**Published:** 2023-05-16

**Authors:** Maggie Lam, Emma Lamanna, Louise Organ, Chantal Donovan, Jane E. Bourke

**Affiliations:** ^1^ Department of Pharmacology, Biomedicine Discovery Institute, Monash University, Clayton, VIC, Australia; ^2^ Centre for Innate Immunity and Infectious Diseases, Hudson Institute of Medical Research, Clayton, VIC, Australia; ^3^ Department of Molecular and Translational Sciences, Monash University, Clayton, VIC, Australia; ^4^ Institut Pasteur, Unit of Antibodies in Therapy and Pathology, INSERM UMR1222, Paris, France; ^5^ School of Life Sciences, Faculty of Science, University of Technology Sydney, Sydney, NSW, Australia; ^6^ Hunter Medical Research Institute and The University of Newcastle, Newcastle, NSW, Australia

**Keywords:** PCLS, airway, intrapulmonary artery, bronchodilator, vasodilator, fibrosis, standardisation

## Abstract

Precision cut lung slices (PCLS) have emerged as powerful experimental tools for respiratory research. Pioneering studies using mouse PCLS to visualize intrapulmonary airway contractility have been extended to pulmonary arteries and for assessment of novel bronchodilators and vasodilators as therapeutics. Additional disease-relevant outcomes, including inflammatory, fibrotic, and regenerative responses, are now routinely measured in PCLS from multiple species, including humans. This review provides an overview of established and innovative uses of PCLS as an intermediary between cellular and organ-based studies and focuses on opportunities to increase their application to investigate mechanisms and therapeutic targets to oppose excessive airway contraction and fibrosis in lung diseases.

## 1 Introduction

Organotypic tissue slices from the lung have been extensively used for metabolic studies and toxicology assays (Freeman and O’Neil, 1984; Fisher et al., 1994; Yilmaz et al., 2019). However, the development of the precision cut lung slice (PCLS) technique, with improved methods for maintaining viability and function in prolonged culture, has resulted in numerous applications beyond these simple global measures of tissue activity and damage.

PCLS are a living tissue preparation, containing all resident cells, including smooth muscle cells, epithelial cells, and fibroblasts. These cells maintain their intercellular interactions and cell-to-matrix relationships within the complex structures of the lung. Pioneering work with PCLS involved the assessment of airway and artery contractility or ciliary activity *in situ* by research groups led by Martin (Martin et al., 1996) and Sanderson (Perez and Sanderson, 2005a; Perez and Sanderson, 2005b; Delmotte and Sanderson, 2006). Recent single-cell sequencing analyses have also confirmed the preservation of innate and adaptive immune cells in PCLS (Winters et al., 2021).

PCLS are now widely used to measure integrated cellular responses initiated by inflammatory, fibrotic or infectious stimuli, and for studies of lung damage and regeneration. Importantly, multiple PCLS can be obtained from patient biobanks well as from animal models with established chronic disease, providing for higher throughout and improved translation potential, while minimizing the ethical implications of animal use for *in vivo* studies. Ongoing optimization of long-term culture and preservation methods and the emerging broader applications of PCLS, including their amenability to human omics studies, further emphasizes the unique potential of utilizing PCLS for both basic biology and drug discovery, bridging the gap between cell culture systems and *in vivo* systems.

There have been recent comprehensive reviews on PCLS, focusing on human disease and animal models of disease, including viral and bacterial infections (Liu et al., 2019; Alsafadi et al., 2020; Viana et al., 2022). In this review, we focus on the preparation and preservation of PCLS, measurements of airway and artery smooth muscle responsiveness, airway cilia activity and fibrosis, as well as assessment of dilator and anti-fibrotic treatments.

## 2 Preparation, culture and applications of PCLS

**FIGURE 1:**
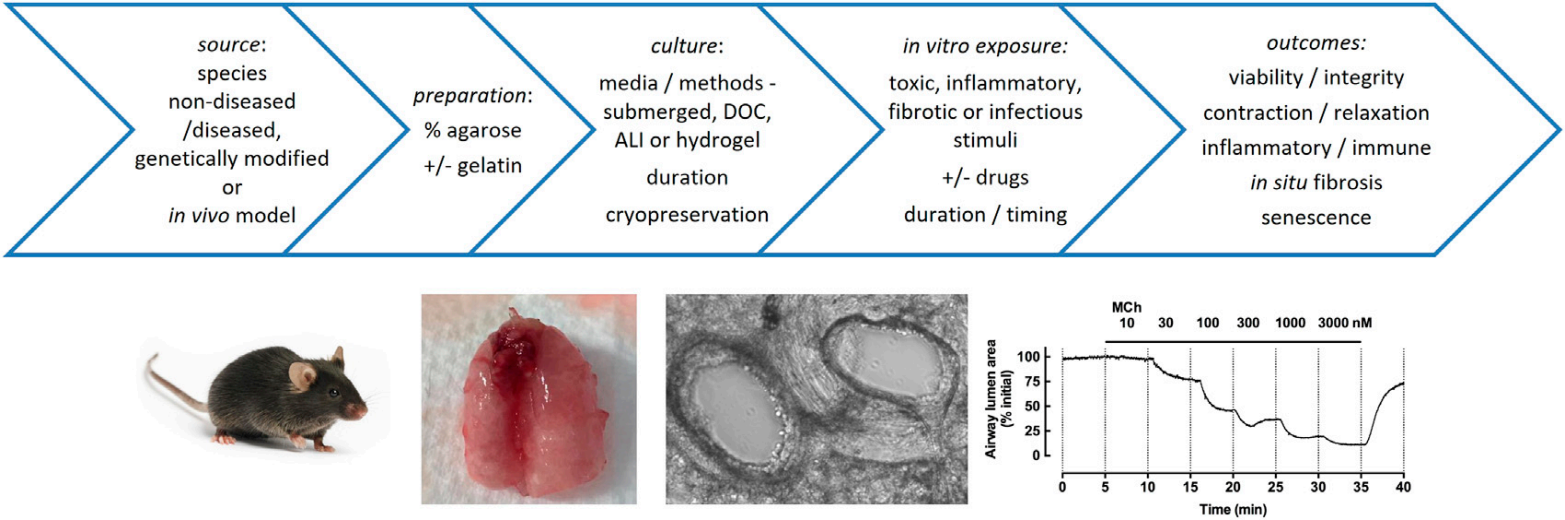
Factors in the preparation, culture and applications of PCLS. Top: Multiple precision cut lung slices can be prepared from non-diseased or diseased agarose-inflated whole lungs, lobes or resections from different species, and cultured under various disease-relevant conditions to test the effects of drugs on acute and chronic tissue responses, extending from airway reactivity to cytokine release to immune responses to infection to deposition of extracellular matrix. Bottom: PCLS have been widely used for assessment of airway contraction. Mouse lungs are shown after inflation of airways with agarose via the trachea and infusion of the pulmonary circulation with gelatin via the right ventricle; phase-contrast image of mouse PCLS shows patent adjacent airway and artery (∼250 μm diameter); trace shows reversible airway contraction to methacholine (MCh) in mouse PCLS, with MCh administered at increasing concentrations at 5 min intervals, and contraction reaching near complete closure of the airway lumen (based on quantitation of images captured every 2 s, normalized to initial area).

Multiple reviews and methodological papers have provided detailed overviews that address many of the factors that need to be considered in the preparation, culture and use of PCLS for various experimental outcomes (Sanderson, 2011; Liu et al., 2019; Alsafadi et al., 2020) (Figure 1). Multiple matched PCLS can be treated with disease-relevant stimuli *in vitro* to mimic the initiation of disease, either in the absence or presence of drugs. PCLS can also be prepared from animal models of disease, from genetically-modified mice or from lung tissue from human subjects where disease is already established, before *ex vivo* drug treatments are applied. As such, both preventative and therapeutic strategies can potentially be tested in PCLS.

### 2.1 Preparation

In the preparation of PCLS for all applications, lungs are inflated using an appropriate volume of liquefied low-melting point agarose solution in physiological buffer, prepared as a 0.5%–3% w/v solution depending on the tissue source. It is important that the agarose concentration used remains consistent within an experiment and is stated in the methods to allow for comparisons between studies performed in different laboratories. The concentration of agarose is important as too low a percentage or alternative mediums that are soft do not allow for sufficient inherent recoil capacity to mediate airway relaxation after contraction (Sanderson, 2011). Agarose is retained within the alveoli after preparation of PCLS, and although this does not prevent airway contraction, it is likely to alter the parenchymal stiffness within a PCLS and should be considered when assessing lung mechanics (Ma et al., 2013).

The lungs are then cooled to below 25°C to set the agarose and stiffen the parenchyma prior to slicing. Inflated lungs can then be sliced with “precision” using a vibratome/compresstome to obtain multiple PCLS with a consistency that was not achievable with the earliest hand-cut lung slices (Placke and Fisher, 1987; Dandurand et al., 1993). PCLS thicknesses vary depending on application, with slices typically ranging from 150 to 300 μm for optimal imaging of contractile responses, or up to 500 μm, if being fixed and sectioned for staining or to obtain sufficient yield of RNA (Sanderson, 2011; Alsafadi et al., 2017; Niehof et al., 2017; Stegmayr et al., 2021).

The instillation of agarose through to the parenchyma can occur via the trachea for relatively small species, such as rodents (Dandurand et al., 1993; Martin et al., 1996), while individual lobes of larger species, such as sheep or human, can be inflated via the bronchi (Wohlsen et al., 2003; Lambermont et al., 2014). Although human surgical resection samples are more readily available than intact lung lobes, preparation of PCLS from these samples may be more challenging. If the distal airways that terminate in alveoli are not intact, agarose delivered via a segmental airway may leak. For these samples, multiple small airways are cannulated to deliver agarose, while others are clamped to limit leakage (Ressmeyer et al., 2010; Gerckens et al., 2019). Alternatively, agarose can be injected directly into the alveolar tissue with a fine gauge needle to inflate localized regions for slicing (Sturton et al., 2008; Yilmaz et al., 2019).

For airway contraction studies of human and mouse PCLS, a standardized approach of agarose inflation, ideally followed by a bolus of air, is required to obtain PCLS with patent intact airways. The presence of beating cilia lining the airways provides a visible marker consistent with airway viability prior to assessment of contractile responses (Bai and Ai, 2022; Koziol-White, 2022). It should be noted that the preparation of guinea pig PCLS requires the addition of the bronchodilator isoprenaline to both the agarose solution and the initial incubation medium to avoid *post-mortem* airway contraction (Ressmeyer et al., 2006).

Even though intrapulmonary arteries are commonly located adjacent to airways, the preparation of PCLS with patent arteries is challenging. The arteries tend to collapse during agarose inflation, as they are less securely tethered and tear away from surrounding connective tissue. To overcome this artefact, warm gelatin can be perfused via the heart through the pulmonary circulation to provide support for the arteries during the subsequent agarose inflation of the airways. The gelatin dissolves in cultured PCLS to allow subsequent visualization of artery contraction responses *in situ* (Perez and Sanderson, 2005a; Bai and Ai, 2022).

### 2.2 Culture conditions

Protocols for the preparation and subsequent culture of PCLS vary in terms of media conditions and duration, depending on the outcomes of interest (Figure 1). PCLS are subjected to wash protocols to remove cell debris and mediators released during slicing, then maintained in culture, irrespective of the outcome to be measured. Immersion of PCLS in media remains the most convenient and widely used tissue culture method, although the use of particular types of media and supplements to maintain viability and specific functionalities has not always been clearly justified (Sanderson, 2011). While PCLS contain resident structural and immune cells, they lack a circulating blood supply. While this provides for a more controlled environment to assess PCLS responses, it may also be considered a limitation e.g., for studies assessing effects of *in vitro* exposure to allergens or infectious agents. The development of integrated platforms that supply circulating blood-borne cells and other factors and measure their influence on contractile and secretory responses of PCLS will be another step supporting the clinical relevance of this already innovative methodology. Novel approaches to optimize PCLS culture and extend longevity using different media supplements, culture methods (dynamic organ roller culture (DOC), air-liquid interface (ALI) culture and hydrogels) and cryopreservation are detailed later.

### 2.3 Applications and considerations

PCLS are used to investigate multiple disease-relevant processes in addition to airway and artery reactivity.

Human PCLS with parenchyma only, prepared by coring of agarose-inflated lung, are increasingly used in a model of *in vitro* fibrogenesis. This protocol involves a 5-day treatment with a fibrotic cocktail (FC), comprising transforming growth factor-β (TGF-β), tumor necrosis factor-α (TNF-α), platelet-derived growth factor-AB (PDGF-AB) and lysophosphatidic acid (LPA) (Alsafadi et al., 2017). The interested reader is directed to detailed protocols and an extensive troubleshooting guide for the preparation of mouse, sheep and human PCLS used for infection studies, outlining approaches for measuring viability, viral load and iinflammatory responses (Rosales Gerpe et al., 2018; Sewald and Danov, 2022).

A recent methods chapter on mouse PCLS culture provides an excellent overview of both preparation and protocols for numerous useful experimental outputs including imaging for contractility studies, collagen and elastin imaging, mean linear intercept (MLI) measurement of alveolar airspace size, immunofluorescent staining and preparation of decellularized PCLS (Wu et al., 2019). A separate study in rabbit PCLS supports their use for some histomorphometric results such as tissue density and alveolar septal thickness, but suggests that MLI and septal may be confounded by agar infusion (Ragionieri et al., 2023).

In addition, two recently published methods have addressed the challenge of obtaining high-quality RNA from PCLS due to the presence of agarose. Recent methodology advances have also allowed PCLS to be utilized for genetic manipulation and adenovirus transfection (Rosales Gerpe et al., 2018; Ruigrok et al., 2018). Improved isolation of RNA suitable for RTqPCR and RNA microarray analysis has been achieved using a magnetic bead-based isolation approach from PCLS containing 1.5% agarose prepared from human, rat, mice, marmoset and rhesus macaque lungs (Niehof et al., 2017). This protocol for RNA isolation from PCLS has been modified to enable miRNA readouts (Niehof et al. 2021). An alternative protocol obtaining high RNA yields and purity from mouse, pig and human PCLS containing higher agarose concentrations of 3% has been validated for RNA-seq (Stegmayr et al., 2021).

Whether standardization of these various protocols for culture, treatment and measurement of outcomes is required to facilitate prolonged PCLS viability and reproducibility of outcomes is a subject of continued discussion among researchers in the field (Patel et al., 2021; Liu et al., 2022; Preuß et al., 2022). Nevertheless, it is clear that significant insights can be gained using PCLS prepared from experimental animals and humans, extending beyond than cell-based studies. Their scalability compared to *in vivo* studies has led to PCLS becoming more widely adopted as an experimental tool. Of note, a PubMed search for the term “precision cut lung slices” was associated with fewer than 20 publications during the 1990s, increasing to over 400 publications since 2010.

## 3 Investigations of smooth muscle responses and cilia activity in PCLS

Because of the contributions of intrapulmonary airways and arteries to airflow and pulmonary artery pressure respectively, it has been critical to characterize mechanisms regulating their reactivity in health and disease. Traditional organ bath and myography approaches to measure changes in force are limited to readily dissected rings of trachea and bronchi, or main pulmonary arteries and first or second order branches. In contrast, PCLS provide access to relatively inaccessible small airways and arteries (typically 50–500 μm diameter), allowing contraction and relaxation to be visualized under phase contrast while the interdependency of smooth muscle and the surrounding parenchyma is maintained (Sanderson, 2011). While assessment of cilia function is routinely performed using epithelial cells isolated from the large cartilaginous airways, PCLS offer the opportunity for direct measurement of cilia beat frequency (CBF) *in situ* in the disease-relevant small airways*,* providing a more physiological setting to assess their critical role in airway protection.

### 3.1 Airways

One of the most valuable applications of PCLS has been to define the pharmacology and physiology of small airways. Pioneering studies established differential responses to specific contractile agonists with airway size within species, and to different agonists (acetylcholine (ACh), histamine, endothelin-1 (ET-1), serotonin (5-HT) and the thromboxane mimetic U46619) between species including mice, sheep, horses, non-human primates and humans (Martin et al., 1996; Held et al., 1999; Martin et al., 2000; Bergner and Sanderson, 2002; Bergner and Sanderson, 2003; Bai and Sanderson, 2006a; Ressmeyer et al., 2006; Vietmeier et al., 2007; Bai et al., 2009; Bai and Sanderson, 2009; Seehase et al., 2011; Lambermont et al., 2014).

Mechanistic insights into contractile signaling pathways have also been obtained using PCLS. Under confocal microscopy, agonist-induced increases in Ca^2+^ oscillations, occurring via release/uptake from intracellular stores within individual smooth muscle cells, can be visualized in PCLS loaded with Ca^2+^-sensitive fluorescent dyes (Perez and Sanderson, 2005a; Perez and Sanderson, 2005b). The critical role of inositol 1,4,5 trisphosphate (IP_3_) in Ca^2+^ release has been confirmed using flash photolysis of caged IP_3_ to activate IP_3_ receptors leading to airway contraction (Bai et al., 2009). PCLS have also been used to explore the contributions of a parallel pathway, termed Ca^2+^ sensitivity, to sustained airway contraction. Using a pharmacological approach, the application of caffeine and ryanodine to PCLS clamps free intracellular Ca^2+^. Contraction can then occur in the absence of Ca^2+^ oscillations, due to increased Ca^2+^ sensitivity through protein kinase C (PKC) and Rho-activated kinase (ROCK) pathways (Bai and Sanderson, 2006b). These elegant approaches were developed and applied by the late Mike Sanderson, who trained many early career investigators still using PCLS to advance respiratory research.

More recently, a novel platform called tissue traction microscopy (TTM) has been applied to PCLS prepared from agarose-inflated lungs in the standard way then adhered to an adhesive and compliant silicone substrate. TTM provides spatial measurements of contractile force generated by airway smooth muscle within PCLS. Compared with the conventional measurements of changes in airway lumen area, this specialized approach resulted in increased sensitivity and less variability in responses to bronchoconstrictor stimuli in both porcine and human PCLS, and potentially addresses one of the limitations of PCLS by allowing the effects of cyclic stretch to be determined (Ram-Mohan et al., 2020). The wider application of TTM is likely to yield novel insights into the regulation of intrapulmonary airway contractility and responsiveness to dilator drugs.

The assessment of altered airway responses in PCLS in the context of acute infection and chronic lung diseases (asthma, COPD and IPF) is described later. These extensive studies have provided insights into mechanisms underlying altered reactivity to endogenous constrictors and impaired responsiveness to dilators in clinical use, as well as the assessment of assessing alternative therapeutics.

### 3.2 Arteries

Pharmacological assessment of intrapulmonary arteries in PCLS remains comparatively limited compared to airways, and has yet to be widely applied to PCLS prepared from models of pulmonary hypertension (PH). Nevertheless, studies using mouse, rat, guinea pig and human PCLS have established ET-1 as the most potent vasoconstrictor compared to 5-HT, potassium chloride (KCl), adrenaline, noradrenaline, phenylephrine and U46619 (Shi et al., 1998; Held et al., 1999; Moreno et al., 2006; Ressmeyer et al., 2006; Faro et al., 2007; Perez-Zoghbi and Sanderson, 2007; Rieg et al., 2011). Using the approaches applied for assessment of Ca^2+^ signaling in airways, Perez demonstrated that contraction of vascular smooth muscle cells in intrapulmonary arteries in mouse PCLS to 5-HT and ET-1 is also regulated by the frequency of Ca^2+^ oscillations and by Ca^2+^ sensitization (Perez and Sanderson, 2005a; Perez-Zoghbi and Sanderson, 2007). Physiological responses to hypoxia have also been established in PCLS. Sustained hypoxic vasoconstriction of intra-acinar arteries (20–150 μm diameter) has been established and linked to hypoxia-induced ROS production (Paddenberg et al., 2006; Paddenberg et al., 2014).

Several studies have applied disease models to PCLS for assessment of intrapulmonary arteries. PCLS prepared from non-smoker lung tissue before exposure to cigarette smoke extract (CSE) for 24 h, showed increased expression of ET-1 receptors, ET_A_ and ET_B_, and ET-1-induced artery contraction (Milara et al., 2012). Similarly, PCLS prepared from guinea pigs after 2 weeks of daily exposure to cigarette smoke showed increased vasoconstrictor sensitivity to ET-1, along with impaired endothelial-dependent NO production and ACh-mediated relaxation compared with controls (Wright and Churg, 2008).

A single study using PCLS prepared from control subjects and patients with either idiopathic pulmonary fibrosis (IPF) or IPF and PH (IPF + PH) has implicated Janus kinase type 2 (JAK2) as a novel target to oppose contraction of intrapulmonary arteries (Milara et al., 2018). This extensive study demonstrated that the JAK2 inhibitor JSI-124 reduced basal tone, relaxed pulmonary arteries pre-contracted with 5-HT and inhibited the development of 5-HT induced contraction, with the latter protocol consistent with the prophylactic use of dilators in *p*H. Pharmacological approaches suggested that relaxation was mediated by activation of large conductance calcium-activated potassium channel (BKCa) reducing intracellular Ca^2+^. Of note, the responses to JSI-124 were reduced in IPF and IPP + PH PCLS compared to controls, suggesting that JAK2, a non-receptor tyrosine kinase activated by a broad spectrum of vasoactive mediators, might be upregulated in disease (Milara et al., 2018). Further studies are required to identify additional targets regulating vascular responses in PCLS from relevant disease models, and to use PCLS to compare responsiveness to both current and novel vasodilator treatments for PH.

### 3.3 Cilia

Measurement of cilia beat frequency (CBF) using differential interference contrast microscopy and high-speed video recording can provide key insights into mucociliary function in the distal lung. The presence of actively beating cilia activity visualized in PCLS may also be an indicator of airway viability, although these functions may be maintained or lost independently.

Measurements of CBF has shown that it occurs at a faster rate within mouse lung slices than in tracheal rings. Unlike the larger airways, CBF was not increased by extracellular ATP despite increased intracellular Ca^2+^ in the ciliated cells, suggesting constant vigilance for the immediate clearance of foreign material reaching the distal lung (Delmotte and Sanderson, 2006).

While not a major focus of this review, the pathological consequences of reduced CBF have also been explored using PCLS. Short-term *in vitro* treatment of mice PCLS with 2% NaCl was used to cause reversible ciliostasis, resulting in an increased viral yield with subsequent swine influenza virus infection, demonstrating reduced protection when cilia function was compromised (Kurosawa et al., 1995). In addition, while *in vivo* studies have shown variable effects of cigarette smoke and alcohol on CBF, co-exposure of mice PCLS *in vitro* resulted in decreased cilia beating, consistent with the significant decrease in bacterial clearance from the lung observed in an *in vivo* rodent model (Vander Top et al., 2005; Wyatt et al., 2012). Exposure of mouse PCLS to e-cigarette condensates prepared from menthol- and nicotine-containing e-fluids resulted in generalized oxidative stress and cytotoxicity as well as structural and functional consequences on airways, with impaired MCh-induced contractile responses and epithelial damage associated with reduced CBF (Herbert et al., 2023).

## 4 Investigations of airway reactivity and fibrosis in PCLS

Increased airway contraction, loss of bronchodilator sensitivity and fibrosis are common features of many chronic lung diseases. Short-term exposure of PCLS to disease-relevant conditions has the potential to both directly and indirectly influence airway reactivity and dilator responsiveness, and *in vitro* models of fibrogenesis in PCLS are now established. While access to human lung tissue with established disease may be limited, PCLS from *in vivo* models can be prepared to assess therapeutic interventions targeting excessive contraction and established fibrosis for clinical translation. The focus of this section of the review is to highlight knowledge gains from these studies and their impact in identifying therapeutic targets or novel treatments opposing airway contraction and fibrosis.

### 4.1 Assessment of contraction and dilator responsiveness in PCLS

β_2_-adrenoceptor agonists, either alone or in combination with anti-inflammatory steroids, remain the most common treatment for acute asthma exacerbations caused by excessive bronchoconstriction. The effects of these dilator drugs on the signaling pathways leading to airway contraction have been established in human PCLS, with activation of adenylate cyclase/cAMP/PKA pathways to oppose contractile agonist-induced Ca^2+^ oscillations and increased Ca^2+^ sensitivity (Bai et al., 2006a; Delmotte and Sanderson, 2008; Delmotte et al., 2010) (Table 1).

**TABLE 1:** Summary of studies assessing the efficacy and mechanisms of action of bronchodilator agents in PCLS.

Drug class	Bronchodilator	Pre-contraction	Species	References
β-adrenoceptor agonist	Isoprenaline (non-selective)	MCh CCh	Mouse, Rat Human	Bai and Sanderson (2006a), Bai and Sanderson (2006b), Ghosh et al. (2016), Lam et al. (2016)
	Salbutamol (SABA)	MCh	Mouse	Delmotte and Sanderson (2008)
	Formoterol (LABA)	MCh CCh, Histamine	Mouse Human	Delmotte and Sanderson (2010), Cooper et al. (2011)
	Salmeterol (LABA)	CCh	Human	Cooper et al. (2011)
	Indacaterol (ultra-LABA)	5-HT, CCh	Rat, Human	Sturton et al. (2008)
Prostaglandin	PGE_2_	MCh	Mouse	FitzPatrick et al. (2014)
cAMP mimetic	8-Br-cAMP	MCh	Mouse	Bai and Sanderson (2006a), Bai and Sanderson (2006b)
Adenylate cyclase activator	Forskolin	MCh	Mouse	Bai and Sanderson (2006a), Bai and Sanderson (2006b)
Nitric oxide donor	NOC-5	5-HT	Mouse	{Perez-Zoghbi et al. (2010) #10}
Guanylate cyclase activator	BAY 41–2,272, BAY 60–2,770	CCh	Mouse, Human	Ghosh et al. (2016), Koziol-White et al. (2020)
Phosphodiesterase inhibitor	IBMX, caffeine (non-selective)	MCh	Mouse	Bai and Sanderson (2006a), Bai and Sanderson (2006b)
	CC3 (PDE4)	MCh	Rat	Martin et al. (2002)
	Osthole (PDE4D)^a^	ACh	Mouse	Wang et al. (2020)
Protein kinase C inhibitor	GF-109203X	MCh	Mouse	Bai and Sanderson (2006a), Bai and Sanderson (2006b)
Rho kinase inhibitor	Y-27632	MCh	Mouse	Bai and Sanderson (2006a), Bai and Sanderson (2006b)
PPARγ agonist	Rosiglitazone, Ciglitazone^1,^ ^a^ Rosiglitazone^1^ ^a^	MCh, 5-HT, ET-1 MCh	Mouse Rat	Bourke et al. (2014), Donovan et al. (2014), Donovan et al. (2015a), Lam et al. (2016)
Bitter taste receptor (TAS2R) agonist	Chloroquine^a^ Chloroquine, Quinine^a^	CCh MCh	Human Mouse	Deshpande et al. (2010), An et al. (2012) Donovan et al., 2014; Tan and Sanderson, (2014); Rosner et al. (2014)
FFA4 agonist	TUG-891, TUG-1197	CCh	Mouse, Human	Prihandoko et al. (2020)
RXFP1 receptor	Relaxin^b^	MCh, ET-1	Rat	Lam et al. (2016)
GABA PAM	SH-053–2′F-R-CH_3_, MIDD0301	MCh	Mouse	Gallos et al., 2015; Yocum et al., 2019
CaSR NAM	NPS2143	ACh	Mouse	Yarova et al., 2015; Diao et al., 2021

ACh, Acetylcholine; 8-Br-cAMP, 8-bromo-adenosine 3′,5′-cyclic monophosphate; CaSR, calcium sensing receptor; CCh, Carbachol; ET-1, Endothelin-1; FFA4 Free fatty acid receptor four; GABA, Gamma-aminobutyric acid; 5-HT, serotonin; LABA, Long-acting *ß*-adrenoceptor agonist; MCh, Methacholine; NAM, negative allosteric modulator; NO, nitric oxide; PAM, positive allosteric modulator; PPARγ, Peroxisome proliferator activated receptor γ; RXFP1 Relaxin family peptide receptor one; SABA, Short-acting *ß*-adrenoceptor agonist; sGC, soluble Guanylate cyclase; TAS2R taste receptor type 2 efficacy:^1^ in PCLS, from disease model.

^a^ Under conditions of reduced responsiveness to *ß*-agonists.

^b^ Potentiated response to *ß*-agonists.

The capacity of β_2_-adrenoceptor agonists to dilate maximally contracted airways is limited by functional antagonism, even in healthy airways. Efficacy may be further compromised in severe disease, when airways hyperresponsiveness (AHR) is present and repeated use of high doses of dilator therapy can lead to receptor desensitization and loss of sensitivity. Other factors such as smoking and infections are also known to reduce dilator responsiveness by increasing airway inflammation and downregulating β_2_-adrenoceptor expression.

These factors that limit effective dilator responses can be modelled in PCLS, providing a suitable platform for relatively high through-put assessment of potential alternative therapies. As the level of precontraction of airways in PCLS is increased e.g. with higher concentrations of a cholinergic agonist such as MCh, the relaxation response to a β_2_-adrenoceptor agonist such as salbutamol declines. Overnight treatment of PCLS with high concentrations of β_2_-adrenoceptor agonists also abolishes their dilator response (Cooper et al., 2011; An et al., 2012; Bourke et al., 2014; Donovan et al., 2015a; Koziol-White et al., 2020).

To date, relatively few studies have assessed airway contraction and dilator responses in PCLS from *in vitro* or *in vivo* experimental models. In terms of contractile responses, *in vitro* exposure of human PCLS to IL-13, but not to poly I:C, increased contraction to MCh, despite the viral mimetic causing the release of inflammatory cytokines predicted to alter airway reactivity (Cooper et al., 2009). MCh-induced contraction was also unaffected in PCLS from a mouse model chronic allergen challenge, despite established *in vivo* AHR to MCh (Donovan et al., 2013). However, contraction to MCh was altered in PCLS prepared after *in vivo* exposure to cigarette smoke either alone or in combination with flu infection (Donovan et al., 2015b; Donovan et al., 2016). Notably, the dilator potency and efficacy of salbutamol was also markedly reduced in PCLS from *in vivo* cigarette smoke models with and without infection, and shown to be associated with decreased β_2_-adrenoceptor mRNA expression (Donovan et al., 2016).

The effects of numerous other drugs activating adenylate cyclase/cAMP/PKA and guanylate cyclase/cGMP/PKG and other signaling pathways have identified diverse mechanisms leading to airway relaxation (Table 1). While some of the drugs tested are purely experimental tools, positive findings with several drug classes have created considerable interest in their potential as novel therapies.

Recent findings with drugs that interact with soluble guanylate cyclase (sGC) in airway smooth muscle in PCLS are notable, as an increase cGMP would provide an alternative intracellular pathway to the cAMP-dependent relaxation activated by β_2_-adrenoceptor agonists (Ghosh et al., 2016; Koziol-White et al., 2020; Lam and Bourke, 2020). sGC stimulator and activator drugs, BAY 41–2,272 and BAY 60–2,270, had similar efficacy and potency to the long-acting β_2_-adrenoceptor agonist formoterol in human PCLS, with no cross-talk to the adenylate cyclase pathway. Notably, these drugs remained efficacious under conditions of β_2_-adrenoceptor desensitization when relaxation to formoterol was abolished (Koziol-White et al., 2020).

Theophylline, a non-selective inhibition of phosphodiesterase (PDE), is a well-established bronchodilator, more selective inhibitors may be required minimize off-target effects. The mRNA transcripts of all PDE4D isoforms have been detected in the lung (Richter et al., 2005). Of interest, the PDE4D inhibitor osthole relaxed preconstricted airways in mouse PCLS, irrespective of β_2_-adrenoceptor desensitization, by binding to enzyme’s catalytic site to prevent cAMP binding and hydrolysis. Identification of osthole binding sites on PDE4D will guide further development of bronchodilators that may not cause the tachyphylaxis seen with β_2_-adrenoceptor agonists (Wang et al., 2020).

Several other receptors in addition to β_2_-adrenoceptors have been investigated as targets to limit excessive airway contraction (Table 2). Peroxisome Proliferator Activated Receptor γ (PPARγ), Taste Receptor type 2 (TAS2R), Free Fatty Acid Receptor 4 (FFA4), Relaxin Family Peptide Receptor 1 (RXFP1), Gamma-AminoButyric Acid A Receptor (GABA_A_R) and Calcium Sensing Receptor (CaSR) are all expressed on airway smooth muscle cells in the lung.

**TABLE 2:** Summary of culture conditions for validation of long-term responsiveness of PCLS.

PCLS	Culture conditions			Outcomes	References
Human 600 μm	Media: DMEM/F12 with l-glutamine, HEPES, serum-free			Viability, inflammation LPS-induced cytokine response decreased over time	Temann et al., 2017
	Supplements: 7.5% NaHCO_3_, pen/strep/gentamicin/fungizone, ITS-X				
	Media changed daily, for 14 days				
Rat 250–300 μm	Media: MEM with l-glutamine, HEPES			Viability for 29 days, not improved by FBS	Nußbaum et al., 2022
	Supplements: amino acids and vitamins, pen/strep ± 10% FBS			Contractility maintained for 13–25 days depending on mediator, but reduced relaxation	
	Media changed daily, for 29 days				
Mouse 150 μm	Media: DMEM/F12, serum-free			Contractility improved with insulin, increased contraction with IL-13 maintained	Li et al., 2020
	Supplements: 0.1, 0.5, 1, or 10 μg/ml insulin; antibiotic/antimycotic				
	Media changed every 48 h, for 14 days				
Human 500 μm	Media/Supps (all): antibiotic/antimycotic, l-glutamine, HEPES Hydrocortisone (acclimation only)			Viability, histology, inflammation	Patel et al. (2021)
	Media/Supps:E-199, insulin	Media/Supps: DMEM/F12, HEPES	Media/Supps: RPMI-1640, insulin, HEPES, retinyl acetate, 0.05% FBS	Variable but maintained viability, protein content, cytokine response to LPS or Poly I:C	
	Method: submerged or DOC or ALI				
	Media changed 3 times per week, for up to 4 weeks				
Sheep 300 μm	Maintenance media: DMEM with high glucose			Viability and viral infection	Rosales Gerpe et al., 2018
	Supplements: pen/strep/gentamicin/amphotericin B			3 days acclimatization in culture then >95% vector transduction or virus infection	
	8-Br-cAMP, IBMX, dexamethasone			Live-dead stain or resazurin, >90% viable over 4 weeks	
	Infection media: as above +10% FBS + l-glutamine or keratinocyte factor or hepatocyte factor; Media changed every 48 h, for up to 4 weeks				
Human 400–600 μm	Media: DMEM/F12 Supplements: 0.1% FBS, pen/strep			Fibrosis early fibrosis-like changes– fibrotic genes by 24h, ECM deposition, alveolar epithelial reprogramming	Alsafadi et al., 2017
	Fibrotic cocktail (FC)				
	5 ng/ml TGF-β1, 5 uM PDGF-AB, 10 ng/ml TNF-α, 5 uM LPA				
	Media with FC replenished at 48 h, cultured up to 5 days				
Human 250 μm	Media: DMEM/F-12 Supplements: pen/strep amphotericin B			Viability, fibrosis	Khan et al., 2021
	Fibrotic stimulus: 10 ng/ml human TGF-β1, 25 μM MMP inhibitor			Viable and metabolically active; Mesenchymal, epithelial, endothelial and immune cell types surviving for 14 days; conditioned media—induced ECM proteins	
	Media with stimulus replenished at 18 h, then every 72 h for 14 days				
Mice	Hydrogel—encapsulated in PEGNB with PEG-dithiol cross-linker			Viability, tissue integrity	Bailey et al., 2020
	Media: DMEM/F12. Supplements: 0.1% FCS pen/strep, amphotericin B			Viability, PCLS architecture cellular phenotype and vimentin expression > non-encapsulated PCLS	
	Media changed every 48 h, for 21 days				
Human 200–300 μm	Media: DMEM/F-12 Supplements: pen/strep			Viability, tissue integrity and transcriptome	Preuß et al., 2022
	Media changed regularly, up to 4 weeks			Progressive loss of vascular, cilia then alveolar integrity over 2 weeks; epithelium preserved for 4 weeks	

ALI, air–liquid interface; 8-Br-cAMP, 8-bromo-adenosine 3′,5′-cyclic monophosphate; DMEM, Dulbecco’s Modified Eagle Medium; DMSO, dimethyl sulphoxide; FBS, foetal bovine serum; DOC, dynamic organ roller culture; ECM, extracellular matrix; 3-IBMX, isobutyl-1-methylxanthine; LPA, lysophosphatidic acid; MMP, matrix metalloproteinase (MMP); O/N overnight; PDGF-AB, Platelet-derived growth factor-AB; TGF-β, transforming growth factor-β; TNF-α, tumor necrosis factor-α

Apart from CaSR, activation of these receptors using a range of drug-like agonists promotes airway relaxation in PCLS from various species, although their downstream signaling pathways have yet to be fully defined. For synthetic FFA4 agonists, relaxation is mediated at least in part by the release of PGE_2_ that subsequently acts on EP_2_ prostanoid receptors (Prihandoko et al., 2020). Direct comparisons of rosiglitazone (RGZ, PPARγ agonist) and chloroquine (TAS2R agonist) with β_2_-adrenoceptor agonists in PCLS studies have shown that dilator responses to both RGZ and chloroquine are maintained in mouse models of allergic airways disease and under conditions of β_2_-adrenoceptor desensitization in mouse and human PCLS respectively (An et al., 2012; Bourke et al., 2014; Donovan et al., 2014; Sharma et al., 2017), Relaxin (RXFP1 agonist) has additive effects with isoprenaline (non-selective β_2_-adrenoceptor agonist) in rat PCLS (Lam et al., 2016). In considering potential for clinical translation, MIDD0301, a positive allosteric modulator (PAM) of GABA_A_R, elicits airway relaxation, but has limited brain distribution, thus eliminating the potential for sedation (Yocum et al., 2019). Activation of CaSR by endogenous ligands such as spermine, or by calcium itself, promotes contraction, so negative allosteric modulators (NAMs) such as NPS2413 that inhibit contraction offer potential therapeutic benefit (Yarova et al., 2015; Diao et al., 2021).

Many of these dilators have other beneficial effects in the lung, independent of their direct effects on airway smooth muscle. Chronic *in vivo* administration of these drugs in models of allergic airways disease that mimic key features of asthma has been shown to reduce airway inflammation, remodeling and/or the development of AHR (Ward et al., 2006; Yarova et al., 2015; Sharma et al., 2017; Prihandoko et al., 2020). To support their clinical translation for asthma, further studies using human PCLS with increased sensitivity to contractile agonists, either from *in vitro* inflammatory models, or from patients with asthma, are required to provide the most relevant setting for validation of these novel dilators compared to current therapies.

### 4.2 Assessment of fibrosis in PCLS

There is increasing interest in the application of PCLS for investigation of disease mechanisms and treatments for idiopathic pulmonary fibrosis (IPF). Current clinical management involves treatment with nintedanib and pirfenidone, antifibrotic drugs that slow disease progression, but do not reverse established fibrosis.

As previously described, disease-relevant stimuli (TGF-β combined with other components to make a “fibrotic cocktail”, FC) can be applied to PCLS to induce fibrogenesis, resulting in accumulation of ECM proteins implicated in long-term fibrosis in the IPF lung. Both low μM concentrations of nintedanib and mM concentrations of pirfenidone reduced expression of extracellular matrix proteins collagen one and fibronectin (COL1A1, FN1) when drugs were added to human PCLS that had been pre-treated with FC for 48 h followed by co-treatment with drugs (Lehmann et al., 2018). These findings in non-diseased PCLS provide evidence of efficacy in preventing the onset and early stages of fibrosis. Although the access to explanted diseased lungs is relatively limited, preparation of PCLS from the healthy margins of smaller surgical resections and the optimization of long-term culture conditions to establish fibrosis may provide opportunities to test interventions to reverse the newly laid down ECM.

To assess whether therapeutic interventions have the potential to reverse established fibrosis requires preparation of PCLS from the gold standard mouse bleomycin model of IPF or from either resections or explanted lungs from patients with established IPF. Validation of feasibility for this approach for screening anti-fibrotic drugs was first obtained using PCLS from an *in vivo* bleomycin model, which showed decreased hydroxyproline levels over 5 days of treatment with caffeine (Tatler et al., 2016).

More recently, it has been confirmed that PCLS from both bleomycin-treated rodents and human retain characteristics of fibrotic disease consistent with or IPF when cultured for up to 5 days, allowing potential reversal of fibrosis to be assessed. Relatively higher expression of fibrosis-related genes for COL1A1, FN1, *a*-smooth muscle actin (ACTA2), and proteins involved in ECM turnover, matrix metalloproteinase 12 and tissue inhibitor of metalloproteinases (MMP12, TIMP1) was shown in PCLS from mice with prior *in vivo* exposure to bleomycin (Lehmann et al., 2018; Cedilak et al., 2019). Similar levels of fibrosis-relevant proteins including *a*-SMA, collagen I and fibronectin were also present in matched PCLS from IPF patients compared over 5 days in culture (Wei et al., 2021).

Several studies have now modeled the clinical scenario in IPF, by testing the effects of nintedanib or pirfenidone on PCLS from bleomycin models or IPF patients (Mercer et al., 2016; Ahangari et al., 2022). In PCLS from the mouse bleomycin model, 3 days of *in vitro* treatment with the ALK5 inhibitor SB525334 or nintedanib resulted in differential inhibition of the expression of fibrosis-related genes, with both drugs decreasing COL1A1, FN1, but only SB525334 inhibiting ACTA2 (Cedilak et al., 2019).

Another study has used PCLS prepared from lungs of bleomycin-treated rats and patient donors with PF, measuring drug effects on levels of specific neoepitope biomarkers of type I, III and VI collagen formation or degradation in conditioned media (Hesse et al., 2022). Differential effects of nintedanib and pirfenidone were seen between species and treatments. Both PRO-C3 and C3M, neoepitopes associated with formation and degradation of type III collagen respectively, were decreased in nintedanib-treated human PCLS, while treatment of rat PCLS led to a reduction in C3M only. Pirfenidone had a marginal effect on PRO-C3 in human PCLS only, and there were no other notable effects of either nintedanib or pirfenidone on the biomarkers for other forms of collagen.

In terms of identification of novel agents for IPF treatment, a small molecule electrophilic nitroalkene, nitro-oleic acid (NO2-OA), reversed key indices of fibrosis in mice PCLS prepared 14 days post-bleomycin (Koudelka et al., 2022). Positive findings showed reductions in COL1A1 and FN1 gene expression, and other markers of myofibroblast differentiation, proliferation and collagen deposition. Inhibition of PGE_2_ metabolism with the 15-PGDH inhibitor SW033291 was also shown to reduce collagen production in PCLS from IPF patients, consistent with the previously reported anti-fibrotic effects of PGE_2_ (Bärnthaler et al., 2020). Neither of these studies made direct comparisons with pirfenidone or nintedanib.

Epigallocatechin gallate (EGCG), an inhibitor of lysyl oxidase-like2 (LOX2) and TGFβ1 signaling, induced collagen I turnover in IPF PCLS (Wei et al., 2021). EGCG decreased collagen I production and crosslinking and increased its degradation via upregulation of MMP1 and downregulation of TIMP1, while neither nintedanib nor pirfenidone regulated these disease-relevant outcomes (Wei et al., 2021).

Overall, PCLS are being increasingly used as a relatively high-throughput screen for anti-fibrotic drugs for IPF and other interstitial lung diseases. To date, these studies have used PCLS prepared from parenchymal tissue, precluding the measurement of potential collagen deposition around the airways or arteries. Given the unmet need for drugs that reverse rather than retard fibrosis in asthma and COPD as well as IPF, further developments in this area that support broader clinical translation are eagerly anticipated.

## 5 Future directions

So what are the major challenges still to be overcome limiting the wider use of PCLS? Due to loss of viability in culture, the majority of PCLS studies have been performed within days of preparation. This limits their application for *in vitro* modeling of complex disease mechanisms or chronic toxicity and compromises the assessment of drugs to reverse established pathological changes. While short culture periods are sufficient for many studies, conditions need to be optimized to limit potential loss of viability and functional capacity over longer periods. Another limitation is that it is not always possible to utilize the large number of PCLS that can be generated from a single lung, whether from an animal model or from a precious clinical sample. A number of studies have addressed these challenges, validating the effects of specific culture conditions and methods on PCLS viability, structure and diverse functions, and assessing the potential for successful cryopreservation (Tables 2, 3).

**TABLE 3:** Summary of cold storage and cryopreservation studies using PCLS.

PCLS	Culture conditions		Timing and outcomes of functional assays	References
Rat 250–300 μm	Cold storage: DMEM/F12 ± high potassium chloride (solution 1) or		Viability, contraction, response to LPS	Tigges et al. (2021)
	chloride-poor, lactobionate-rich analog (solution 2)		Solution 1 superior to solution 2 or standard DMEM/F-12 No contractility in DMEM/F12 alone after 14 days Gradual reduction in cytokine response in all conditions with time	
	3–28 days at 4°C *For contraction, LPS-induced cytokine release*			
	Media: warmed DMEM/F-12			
Mouse 250 μm	Cryopreservation: 10% DMSO in DMEM/F12		Contractility	Rosner et al. (2014)
	single PCLS/cryovial frozen at -80°C		Tested immediately post thaw	
	liquid N_2_ for storage up to 3 months		Airway contraction maintained after cryopreservation and storage	
	Thaw: rapidly in 37°C water bath			
	*For contraction*			
	Media: DMEM/F-12			
	Supplements: kanamycin/pen/strep/amphotericin B			
	Media changed once an hour for 4 h then daily			
Mice, rats	Cryopreservation: 10% DMSO in DMEM/F12		Viability, response to toxin	Watson et al., 2016
	single PCLS/cryovial, frozen at -80°C		Immediately post thaw or up to 3 days later	
	*For tox study*		Decreased viability and metabolic activity	
	Media: DMEM/Ham’s F-12		Similar toxin-induced changes in cell viability, mitochondrial integrity, and glutathione activity	
	Supplements: kanamycin/pen/strep/amphotericin B			
Human 250 μm	Overnight incubation DMEM/F12/antibiotics		Viability, immune cell function, contractility	Bai and Ai (2022)
	Cryopreservation: 10% DMSO in DMEM/F12		Immediately post thaw or up to 6 days later	
	3 PCLS/cryovial in 100% isopropyl alcohol, then frozen O//N		Contraction and relaxation, calcium signaling maintained	
	-80°C liquid N_2_ for long-term storage			
	Thaw: rapidly in 37°C water bath		Cell viability, immune cell functions maintained	
	*For contraction*	*For lymphocyte activation*		
	Media: DMEM/F-12	Media: RPMI		
	Supplements: pen/strep	Supplements: 10% FBS, pen/strep/antimycotics		
		Media changed every 48 h		

DMEM, Dulbecco’s Modified Eagle Medium; DMSO, dimethyl sulphoxide; FBS, foetal bovine serum; DOC, dynamic organ roller culture; 3-IBMX, isobutyl-1-methylxanthine; LPA, lysophosphatidic acid; MMP, matrix metalloproteinase (MMP); O/N overnight; PDGF-AB, Platelet-derived growth factor-AB; TGF-β, transforming growth factor-β; TNF-α, tumor necrosis factor-α

### 5.1 Media supplementation

With daily media changes, PCLS have been reported to maintain normal metabolic activity and overall structural integrity in serum-free media for at least 14 days in serum-free media, although the cytokine response to lipopolysaccharide (LPS) was markedly reduced over time compared to the response in fresh PCLS (Temann et al., 2017). Loss of contractile responses typically occurring within several days in culture*,* so that only short-term culture of PCLS in serum-free media is widely used when functionality of smooth muscle cells is required. Recently, the addition of insulin has been shown to preserve airway contractile responses in mouse PCLS for up to 2 weeks (Li et al., 2020).

Longer-term viability remains a challenge. A recent study cultivated more than 1,500 human PCLS from 16 different donors under standardized, serum-free conditions for up to 28 days. While viability of PCLS was well preserved, transcriptome analysis revealed a significantly increased immune response and significantly decreased metabolic activity within the first 24 h after PCLS generation. A continuous loss of cells was observed over time, occurring at different rates in the different anatomical compartments. The significant loss of vascular integrity within days extended to a gradually decrease in ciliary beat in the small airways after 1 week. Alveolar integrity was preserved for about 2 weeks, while bronchial epithelium was well preserved for 4 weeks (Preuß et al., 2022).

Media supplement requirements for long term *ex vivo* infection studies in sheep PCLS have been detailed, with PCLS maintained in DMEM containing antibiotics and already supplemented with 10% serum and glutamine. The selective addition of recombinant human keratinocyte factor or hepatocyte factor, present only during the infection period of the protocol, supports propagation of the virus of interest before the PCLS are returned to maintenance media, remaining viable for up to 4 weeks (Rosales Gerpe et al., 2018).

### 5.2 Culture methods

A recent study has systematically compared the standard protocol of human PCLS submerged in media with dynamic organ roller culture (DOC) or air-liquid interface (ALI) culture. PCLS could be maintained for up to 28 days in different combinations of each culture method with three different types of media. In general, PCLS showed similar histological features, viability and secretory responses to pro-inflammatory bacterial and viral mimetics LPS and poly I:C, irrespective of media or method (Patel et al., 2021). These findings require confirmation, since the overall numbers of PCLS tested in this study was relatively small.

Poly (ethylene glycol)-based hydrogel platforms have recently been evaluated as an alternative culture method for PCLS (Bailey et al., 2020). When encapsulated, PCLS maintained architecture, viability, cellular phenotype (as measured by SFTPC) and vimentin expression. These results suggest that the extended culture times required to study chronic lung diseases *ex vivo* could be possible using PCLS in hydrogels, validation of *in vitro* disease models or measurement of key disease-relevant outcomes has yet to be presented.

### 5.3 Cryopreservation and cold storage

Cryopreservation is used to preserve the structure and function of intact living cells and tissues. Using the conventional cryopreservative dimethyl sulfoxide (DMSO), PCLS have been frozen, stored and thawed for study of airway reactivity at extended time points (Table 3). Previous studies in mouse PCLS have shown that cryopreservation of slices for up to 2 weeks did not alter airway contraction to MCh or relaxation to the dilator, chloroquine (Rosner et al., 2014). In human freeze-thawed PCLS, airways had similar responses to MCh, histamine, formoterol as never-frozen PCLS, and inhibition of histamine-induced Ca^2+^ oscillations by the bitter taste agonist quinine could be visualized (Bai et al., 2016). Immune functions were also maintained after cryopreservation, shown as phagocytic activity and proliferation of lymphocytes supporting the broad use of cryopreserved PCLS for immunological studies in addition to physiological and pharmacological studies.

In a separate toxicology study, the viability and metabolic activity of cryopreserved PCLS from rats and mice was lower upon thawing than never-frozen PCLS (Watson et al., 2016). However, the expected changes in cell viability, mitochondrial integrity, and glutathione activity when exposed to toxin zinc chloride were maintained, and correlated with lung injury markers in lavage fluid from rats intratracheally instilled with zinc chloride. These findings support the feasibility of also using cryopreserved PCLS for predictive toxicology (Watson et al., 2016).

An alternative method to optimize PCLS utilization without cryopreservation evaluated long-term hypothermic (cold) storage at 4°C, in either DMEM/F-12 or two different preservation solutions (Tigges et al., 2021). After 14 days of cold storage, a range of parameters were better preserved after storage in potassium and chloride-rich tissue preservation solution (solution 1) and a chloride-poor, lactobionate-rich version (solution 2) compared with DMEM/F-12. PCLS stored in solution one responded substantially longer to inflammatory stimulation with LPS, resulting in higher TNF-α levels in conditioned media than in PCLS stored in DMEM/F-12 or solution 2. Notably, bronchoconstriction to ACh was observed after 14 days in both optimized solutions, but not DMEM/F-12 alone (Tigges et al., 2021). This approach may reduce logistical challenges in shipment of PCLS between laboratories and provide opportunities for stockpiling of PCLS samples.

### 5.4 Not just airways, not just fibrosis

Given the diversity of diseases affecting the lung, there are many new and emerging applications for PCLS beyond the scope of this brief review. PCLS can be used to study local immune response and tissue factors influencing infectivity, as well allow integrated assessment of antiviral and anti-inflammatory agents (Liu et al., 2015). There is likely to be an increased focus on the use of PCLS to study lung infection, with emerging knowledge of populations of cells present, and the ongoing impact of the COVID-19 pandemic.

PCLS are providing new approaches to study both lung development and aging. Mice PCLS have been used to study the initiation of alveologenesis (Pieretti et al., 2014), with more recent advances in time-lapse imaging and live cell staining demonstrating that cell migration is a key to long-term alveologenesis during postnatal lung development (PMID: 30862802) (Akram et al., 2019). Increases in airway but not vascular reactivity have been demonstrated in mice PCLS a double-hit *in vivo* model of bronchopulmonary dysplasia (BPD) (Royce et al., 2016; Bui et al., 2019). More recently, PCLS from preterm rabbits were used in an *in vitro* model of BPD, in which septal defects and other structural abnormalities could be induced by hyperoxia (Ragionieri et al., 2023). While recognizing that further refinement may be required to mimic the influence of cyclical stretch from ventilation on development, further studies using PCLS from BPD and other early life models may provide novel insights into pathways involved in septation and identify potential therapeutic targets to enhance alveolarization.

As shown from their application in fibrosis studies, PCLS provide a translational model to study aberrant ECM remodeling and its influence on diverse cellular functions. The range of pathological conditions influenced by the ECM extends to aging (Blokand et al., 2020), but only limited studies have used PCLS to study senescence. Methods for determination of senescent myofibroblasts in PCLS have recently been validated (Cruz et al., 2021) and irradiated PCLS developed as an *ex vivo* model of cellular senescence (Narvaez et al., 2022). This will provide opportunities to study aging-related mechanisms, including bidirectional interactions between the ECM and senescent cells within PCLS.

## 6 Conclusion

PCLS are clearly established as a multifaceted and powerful tool to assess mechanisms contributing to multiple disease-relevant outcomes in the distal lung. Defining the optimum conditions for experimentation is critical, but will vary depending on whether only acute outcomes are being assessed or whether there is a need to establish a disease phenotype *ex vivo*. In general, DMEM/F12 is the medium of choice for PCLS studies, with frequent media changes appearing to support viability and function, and specific supplements added to maintain contractility and to support infection. The encapsulation of PCLS may extend survival for assessment of synthetic responses (cytokine release, ECM production), but functional assessment of contractility or cilia function, which requires airway, arteries and beating cilia to be visualized *in situ*, remains challenging in encapsulated PCLS. Irrespective of their intended use, PCLS offer the potential for relatively high throughput with fewer ethical implications than *in vivo* animal studies. It is critical for both preparation and culture conditions to be reported in detail to enable researchers to reproduce and extend research findings across the diverse applications of PCLS.

Exciting opportunities lie in the application of PCLS exposed to *in vitro* culture conditions to maintain viability and mimic the disease environment, from *in vivo* disease models, and from human diseased lung tissue. Now well established for assessment of novel bronchodilators and anti-fibrotics, further development of the PCLS technique will only enhance its utility to screen both preventative and treatment modalities as a bridge to clinical translation.
